# µ-Conotoxins as Leads in the Development of New Analgesics 

**DOI:** 10.3390/molecules15042825

**Published:** 2010-04-19

**Authors:** Raymond S. Norton

**Affiliations:** Walter and Eliza Hall Institute of Medical Research, Parkville, Victoria 3052, Australia; E-Mail: ray.norton@wehi.edu.au

**Keywords:** cone shell, toxin, sodium channel, pain, peptide mimetic

## Abstract

Voltage-gated sodium channels (VGSCs) contain a specific binding site for a family of cone shell toxins known as µ-conotoxins. As some VGSCs are involved in pain perception and µ-conotoxins are able to block these channels, µ-conotoxins show considerable potential as analgesics. Recent studies have advanced our understanding of the three-dimensional structures and structure-function relationships of the µ-conotoxins, including their interaction with VGSCs. Truncated peptide analogues of the native toxins have been created in which secondary structure elements are stabilized by non-native linkers such as lactam bridges. Ultimately, it would be desirable to capture the favourable analgesic properties of the native toxins, in particular their potency and channel sub-type selectivity, in non-peptide mimetics. Such mimetics would constitute lead compounds in the development of new therapeutics for the treatment of pain.

## 1. Introduction

Voltage-gated sodium channels (VGSCs) play key roles in the electrical excitability of cells by regulating the influx of sodium ions. In mammals, nine different subtypes (Na_V_1.1-1.9) have been identified, each with distinct distributions in the body [1]. Of particular interest are the neuronal subtypes, several of which have been implicated in the perception of pain [2,3]. Modulators of these subtypes therefore may have potential therapeutic applications as analgesics. 

Many peptide toxins have evolved to target VGSCs with high selectivity and potency [1,4]. Three families of conotoxins target voltage-gated sodium channels (VGSCs), causing either inhibition (μ- and μO-conotoxins), delayed inactivation (δ-conotoxins) or channel blockade (μ-conotoxins) [5]. The μ-conotoxins are the only polypeptide toxins known that bind to Site 1 on VGSCs, one of several toxin binding sites identified on these channels [6]. The pore-forming α-subunit of each VGSC consists of four homologous domains, each containing six putative trans-membrane helices S1-S6, with the Na^+^ channel thought to be formed by the S5-S6 loops from all four domains. Site 1, located on the extracellular surface of this pore, binds the guanidinium alkaloids tetrodotoxin (TTX) and saxitoxin (STX) as well as the μ-conotoxins. Not all VGSCs, however, bind TTX; of the nine well-characterized α-subunits cloned to date from mammals [7,8], at least three, Na_V_1.5, Na_V_1.8, and Na_V_1.9, can be classified as TTX-resistant. The binding sites for TTX and μ-conotoxins overlap, but are not identical [9,10,11,12,13]. In this short review, I describe the μ-conotoxins, their structures and structure-function relationships, and attempts to date to truncate the native peptides to generate minimized analogues that retain their VGSC-blocking activity. 

## 2. μ-Conotoxins

**Figure 1:**
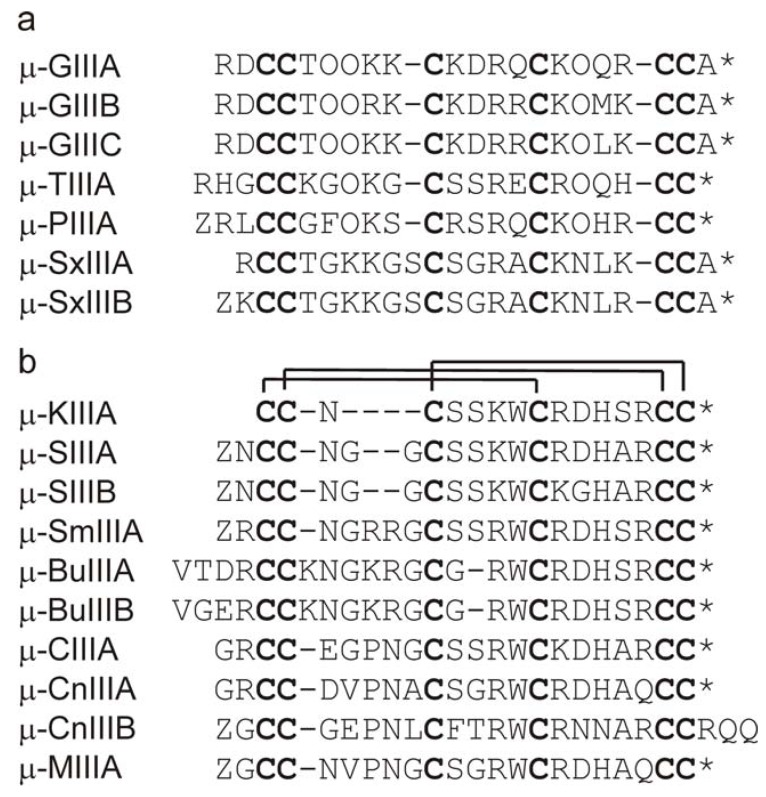
Amino acid sequences of μ-conotoxins from the (a) M-4, and (b) M-5 branches of the M-superfamily [15,16]. Horizontal lines denote the location of disulfide bridges. Z represents pyroglutamate, O *trans*-4-hydroxyproline, and * a C-terminal amide. References for the amino acid sequences are as follows: μ-GIIIA, μ-GIIIB and μ-GIIIC [17], μ-TIIIA [18], μ-PIIIA [19], μ-SxIIIA and μ-SxIIIB [20]; μ-KIIIA and μ-SIIIA [21], μ-SIIIB [22], μ-SmIIIA [23], μ-BuIIIA and μ-BuIIIB [24], μ-CIIIA, μ-CnIIIA, μ-CnIIIB and μ-MIIIA [25]. The sequence of μ-BuIIIC [24] is not shown in this Figure; it contains eight residues in the first loop. The disulfide connectivities shown above the sequences in (b) have been confirmed experimentally for a number of the M-4 and M-5 toxins shown here, but not yet for all.

μ-Conotoxins contain 16-25 residues, with six Cys residues arranged in a class III framework [5,14]. They belong to the M-superfamily of conopeptides, specifically the M-4 and M-5 branches of this superfamily [15,16], which are defined as such based on the number of residues in the third loop, separating the fourth and fifth half-cystine residues (Figure 1). The M-4 branch includes κM- and ψ-conotoxins, which target other classes of ion channel [5,14], as well as μ-conotoxins, whereas the M-5 branch consists exclusively of μ-conotoxins [16]. The μ-conotoxins GIIIA and GIIIB from *Conus geographus* were among the first to be characterized. μ-GIIIA specifically blocks skeletal muscle Na^+^ channels (Na_V_1.4), having significantly lower affinity for other Na^+^ channel subtypes [26,27,28]. μ-GIIIA and μ-GIIIB have a rather complex interaction with the Na^+^ channel pore, with several residues in the toxin contributing to high affinity binding. Arg13 (the conserved Arg in the second inter-cysteine loop) is particularly important [27,29,30] as it occupies the mouth of the channel and inhibits Na^+^ flux by acting as both a steric and electrostatic barrier [12,31,32]. The replacement of numerous other residues of μ-GIIIA affects its function, indicating that many parts of the toxin surface interact with the Na^+^ channel [33,34]. μ-GIIIA therefore differs from the Na^+^ channel-blocking guanidinium toxins, TTX and STX, which appear to occlude the narrow part of the pore [32,35]. 

μ-PIIIA, from the fish-hunting snail *C*. *purpurascens*, also has a strong preference for the skeletal muscle subtype but can block other TTX-sensitive subtypes as well, albeit with lower affinity [19,36,37]. μ-TIIIA from *C. tulipa* was selective for Na_V_1.2 and 1.4 over Na_V_1.3, 1.5, 1.7, and 1.8, and had no effect on rat dorsal root ganglion neuron current. In contrast, μ-SmIIIA, from the fly speck cone snail *C*. *stercusmuscarum*, blocks TTX-resistant VGSCs in frog sympathetic and dorsal root ganglia but has little effect on neuronal TTX-sensitive currents [23]. μ-SmIIIA thus represents the first specific antagonist for TTX-resistant Na^+^ channels (at least in amphibians). 

Since the discovery of μ-SmIIIA, several additional μ-conotoxins have been described that blocked TTX-resistant Na^+^ currents in amphibian DRG neurons [21,38,39]. These conotoxins, isolated from fish-hunting snails such as *C*. *striatus*, *C*. *kinoshitai*, *C*. *magus*, *C*. *consors* and *C*. *catus*, share sequence similarity with μ-SmIIIA in the C-terminal region (Figure 1). They blocked TTX-resistant and TTX-sensitive sodium currents with different potency and selectivity [39]; μ-CnIIIA appeared to discriminate most between the two different types of Na^+^ currents. Some of these μ-conotoxins exhibited potent activity following intracerebroventricular injection in behavioural bioassays in mice, suggesting that they target mammalian CNS Na^+^ channel subtypes [39]. Recently, it was shown that μ-KIIIA blocked several subtypes of mammalian neuronal VGSCs and displayed potent analgesic activity following its systemic administration in mice [39]. 

## 3. Structures and Structure-Function Relationships

**Figure 2:**
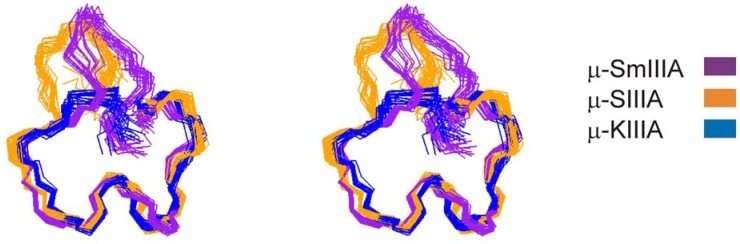
Stereo views of families of 20 final structures of μ-KIIIA (blue) [BMRB accession number 20048] [40], μ-SIIIA (orange) [BMRB accession number 20023] [41] and μ-SmIIIA (purple) [PDB ID 1Q2J] [42], superimposed over backbone heavy atoms (N, C^α^, C’) of residues 4-16 for μ-KIIIA (residues 8-20 for μ-SIIIA and residues 10-22 for μ-SmIIIA).

A superposition of the solution structures of μ-SmIIIA, μ-SIIIA and μ-KIIIA is shown in Figure 2. All three peptides have an essentially identical C-terminal region but differ in the length of the first N-terminal loop (Figure 1) [21]. Consistent with the absence of hydroxyprolines (Figure 1), all three structures consist of a single conformation with all peptide bonds in the *trans* configuration. Comparing these structures confirms that the number of residues in the first loop does not affect the overall conformations of the second and third loops. Superimpositions of the three structures over the backbone heavy atoms of the second and third loops (residues 4-16 of μ-KIIIA) give a mean global pairwise RMSD of 0.92 Å, with μ-KIIIA being more similar to μ-SIIIA (RMSD 0.88 Å) than μ-SmIIIA (RMSD 1.34 Å). As in μ-KIIIA, the secondary structures of μ-SIIIA and μ-SmIIIA are characterized by an α-helix corresponding to residues 7-12 of μ-KIIIA, although in μ-SmIIIA this helix was somewhat distorted. As expected, the main difference lies in the length of the first inter-cysteine loop towards the N-terminal, which, in all three peptides, is oriented away from the C-terminal region (Figure 2).

**Figure 3:**
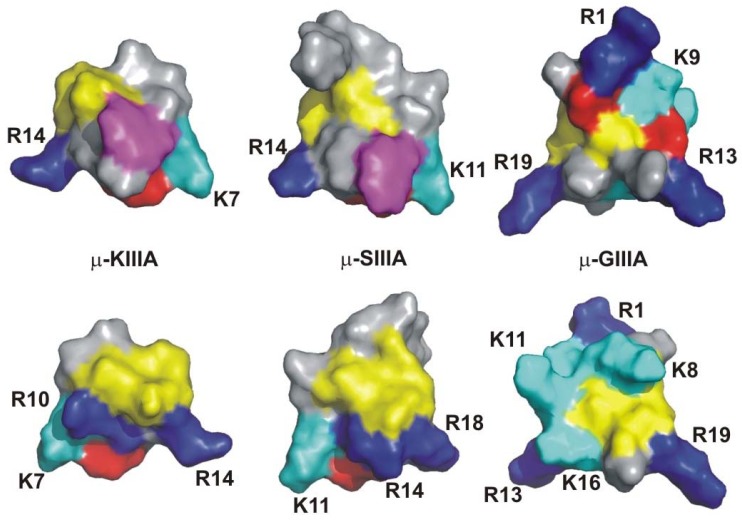
Surface representations of the structures of μ-KIIIA [BMRB accession number 20048] [40], μ-SIIIA [BMRB accession number 20023] [41] and μ-GIIIA [PDB ID 1TCG] [43]. Arg residues are coloured dark blue, Lys cyan, Asp red, Cys yellow, Trp purple and all others grey. The lower views are flipped by 180º around the vertical axis relative to the upper views. Structural figures were prepared using PyMOL (Delano, W.L. The PyMOL Molecular Graphics System, Delano Scientific, San Carlos, CA, USA; 2002 http://www.pymol.org).

Surface representations of the structures of μ-KIIIA, μ-SIIIA and μ-GIIIA are shown in Figure 3. The spatial orientations of several conserved Arg and Lys side chains in μ-KIIIA and μ-SIIIA are similar to those in μ-GIIIA but the N-terminal regions differ, reflecting the all *trans* conformation for μ-KIIIA and μ-SIIIA, as opposed to the *cis* conformation of the peptide bond preceding Hyp7 in μ-GIIIA and μ-GIIIB [43,44,45,46]. The Trp side chain, which is unique to the M-5 group of μ-conotoxins (Figure 1), is a prominent feature of the surface of these molecules shown in the upper views in Figure 3, which also displays fewer charged residues than in μ-GIIIA, and in particular lacks the Asp side chains visible in μ-GIIIA. Indeed, the comparisons in Figure 3 emphasize the similarity to one another of the dispositions of charged residues on the surfaces of μ-KIIIA and μ-SIIIA, and their differences from the surface of μ-GIIIA.

### 3.1. Key Residues of μ-KIIIA Lie on an α-Helix

A study of structure-activity relationships in μ-KIIIA identified key residues important for its activity on the mammalian neuronal Na_V_1.2 and skeletal muscle Na_V_1.4 subtypes, and demonstrated that the engineering of μ-KIIIA could provide subtype-selective therapeutics against mammalian VGSCs for the potential treatment of pain [39]. The structure of μ-KIIIA shows that the key residues important for activity against Na_V_1.2 and Na_V_1.4 [39] all reside on the α-helical region, with the exception of Arg14. This was also the case for μ-SIIIA, suggesting an important role for this α-helix in presenting key residues to mammalian sodium channels [22]. Consistent with this, the Cys1-Cys9 disulfide bond could be removed without significantly distorting the α-helix and with minimal change in the activity of the peptide against the Na_V_1.2 subtype and only slight reductions in affinity for the Na_V_1.4 and Na_V_1.7 subtypes [40]. As the disulfide-deficient analogue maintains activity similar to that of the native toxin, the conformation of the N-terminal region may not be critical in maintaining the structure and activity of the peptide against the Na_V_1.2 subtype. However, the greater flexibility of the N-terminal region in the disulfide-deficient analogue may influence the selectivity profile of the toxin for other Na_V_ subtypes, such as Na_V_1.4 and Na_V_1.7. Interestingly, μ-SIIIA, which has a longer N-terminal loop, reportedly did not target the Na_V_1.1 and Na_V_1.7 subtypes [22]. 

Although there was minimal change in the activity against Na_V_1.2 and Na_V_1.4 upon removal of the Cys1-Cys9 disulfide bridge in μ-KIIIA, disulfide deletion increased both *k*_on_ and *k*_off _of the peptide against these subtypes. Similarly, disulfide deletion also increased the kinetics of peptide binding to its target channel in the case of conkunitzin-S1 [47], a neurotoxin that binds voltage-gated potassium channels. In that study, addition of a disulfide bond to the native toxin decreased both *k*_on_ and *k*_off _for binding of the toxin to the Shaker potassium channel target, but did not affect the overall blocking activity against this channel. Thus, it is possible that the enhanced flexibility of a peptide toxin conferred by removing a conformational restraint such as a disulfide bridge may enhance its ability to both associate with and dissociate from its target channel. It is also possible that the slightly more expanded and flexible N-terminal region of μ-KIIIA[C1A,C9A] compared with μ-KIIIA may enable it to sample a larger volume of conformational space and thereby facilitate eventual binding to the channel. 

The roles of the N-terminal and C-terminal regions have been explored by constructing chimeras of μ-SmIIIA and μ-PIIIA in which their N- and C-terminal halves were recombined [42]. It was evident that residues in the C-terminal half of μ-SmIIIA confer affinity for TTX-resistant VGSCs. Substitution of the last nine residues of μ-PIIIA with the corresponding ten residues from μ-SmIIIA conferred affinity for TTX-resistant VGSCs, whereas substitution of the last ten residues in μ-SmIIIA with the corresponding nine residues from μ-PIIIA abolished μ-SmIIIA’s affinity for TTX-resistant VGSCs. 

Extensive mutational studies have also been carried out on μ-GIIIA. Arg13 is a particularly important residue for binding as well as for blockade of channel function [27,29,30] [it should be noted that Arg13 in μ-GIIIA does not correspond to Arg14 in μ-KIIIA, the equivalent residue in μ-KIIIA being Lys7 (Figure 1)]. When μ-GIIIA is bound, Arg13 appears to occupy the mouth of the channel and inhibits Na^+^ flux by acting as both a steric and electrostatic barrier [12,31,32]. The μ-GIIIA mutant R13Q binds with poor affinity, and, when bound, reduces channel-conductance by only 70% compared to the native toxin which blocks conductance 100% [27]; this has been taken to indicate that the toxin binds to the pore with Arg13 very close to the selectivity filter. Replacement of numerous other residues of this toxin also affects its function, indicating that many parts of its surface interact with the Na^+^ channel [33]. Charges grouped on one side of the toxin at positions 2, 12, and 13 had a weaker influence, whereas residue 16, on the opposite face, was more important. It appears that one side of the toxin is masked by binding to the pore, but Lys16 is exposed to an aqueous cavity accessible to entering ions [34]. As noted by Hui *et al*. [32], μ-GIIIA therefore differs from other ion channel inhibitors such as the charybdotoxin family of K^+^ channel blockers and the Na^+^ channel-blocking guanidinium toxins, which appear to occlude the narrow part of the pore. TTX and STX block at the selectivity filter [35] of a variety of Na^+^ channels.

Recently, Zhang *et al.* [48] reported the unexpected finding that μ-KIIIA and TTX could bind simultaneously to Site 1 and act in concert. μ-KIIIA at saturating concentrations blocked Na_V_1.2 only partially, with a residual current that could be blocked completely by TTX. Moreover, the kinetics of block by TTX and μ-KIIIA were each affected by the prior presence of the other toxin. It was proposed that, in the bi-liganded Na_V_ complex, TTX is bound between the peptide and the selectivity filter, implying that Site 1 may consist of overlapping sub-sites. 

## 4. Voltage-Gated Sodium Channels and Pain

VGSCs play a key role in nociceptive pathways [2]. Using mostly genetic methods, several subtypes of sodium channels, such as Na_V_1.3, 1.7 or 1.8, have been validated as molecular targets for the treatment of pain [49,50]. Na_V_1.9, a TTX-resistant channel restricted to the peripheral nervous system, has also received attention in relation to pain, although it may be more important for inflammatory rather than neuropathic pain [49,51,52]. 

The importance of the TTX-sensitive channel Na_V_1.7 was emphasized by studies of three families from northern Pakistan with an inability to sense pain; mutations in the gene *SCN9A*, encoding the α-subunit of Na_V_1.7, which is strongly expressed in nociceptive neurons, caused loss of function of this channel. These findings imply that Na_V_1.7 is an essential and non-redundant requirement for nociception in humans [53]. Expression of another TTX-sensitive channel, Na_V_1.3, is significantly up-regulated in various neuropathic pain states, including nerve injury, spinal nerve ligation, postherpetic neuralgia and diabetic neuropathy [49,54,55,56,57].

Na_V_1.8 is expressed primarily in the peripheral nervous system, but, unlike Na_V_1.3 and Na_V_1.7, it is resistant to TTX. Selective Na_V_1.8 mRNA axonal transport and local up-regulation may contribute to the hyperexcitability of peripheral nerves in some neuropathic pain states [58]. A potent and selective Na_V_1.8 blocker, A-803467, has been shown to attenuate neuropathic and inflammatory pain in the rat [59], validating this channel subtype as a target for analgesic development.

μ-KIIIA has been shown to block several subtypes of mammalian neuronal VGSCs and displays potent analgesic activity in the formalin test in mice following systemic administration, with an ED_50_ of 0.1 mg/kg (intraperitoneal, bolus injection) [39]. Similarly, μ-SIIIA exhibited analgesic properties in the inflammatory pain assay in mice following systemic administration, with an ED_50_ of 0.9 mg/kg (i.p.) [60]. Introduction of amino-3-oxapentanoic acid as a backbone spacer in μ-SIIIA significantly improved its analgesic properties with respect to both duration of action and potency (ED_50_ 0.05 mg/kg i.p.) [60]. The very recently described μ-conotoxin μ-BuIIIB [24] potently blocks Na_V_1.3, with an IC_50_ of 0.1 μM (Zhang, Yoshikami, *et al*., to be published), in contrast to μ-KIIIA and μ-SIIIA, which are potent blockers of other VGSCs but not Na_V_1.3 [39,41]. μ-BuIIIB should therefore serve as a valuable tool to evaluate the contribution of the Na_V_1.3 subtype to various pain states. Thus, μ-conotoxins target neuronal subtypes of sodium channels that are important in pain perception. While most μ-conotoxins characterized to date lack the Na_V_1 subtype specificity desirable for therapeutic development (indeed, in many cases their selectivity has not been fully defined), there is every reason to believe that suitably selective analogues can be engineered. Such subtype-selective μ-conotoxin analogues will not only be valuable leads in the search for new analgesics but also valuable tools for dissecting the roles of individual Na_V_ subtypes in pain perception.

## 5. Exploiting the Therapeutic Potential of μ-Conotoxins

### 5.1. Peptides as Drugs

Peptides typically display high potency and target selectivity, making them valuable leads in the development of new therapeutics. Indeed, many peptides have made the transition to pharmaceutical products [61,62], including cyclosporine [63], somatostatin analogues [64,65], glatiramer acetate [66], exenatide [67] and the cone shell toxin ziconotide [68]. Nonetheless, converting lead peptides to drugs represents a considerable challenge. Many peptides lack oral bioavailability as a consequence of their susceptibility to proteolysis in the gut, inefficient transport across the intestinal wall, and proteolytic degradation in the bloodstream [69].

Peptides are also susceptible to rapid renal clearance, as illustrated by the 35-residue polypeptide ShK toxin. This peptide, and analogues thereof [70], are potent immunosuppressants that are of interest as therapeutic leads for the treatment of multiple sclerosis and other autoimmune diseases [71]. One analogue of ShK composed entirely of D-amino acids retained activity but was resistant to proteolysis [72]. However, the circulating half-life of D-allo-ShK was only slightly longer than that of ShK, implying that renal clearance was the major determinant of its plasma level. One potential strategy to circumvent this problem is to encapsulate the peptide in a slow-release formulation that provides predictable rates of release into the bloodstream [73,74] as well as protection from peptidases and proteases. Another approach to prolong plasma half-life would be to couple the peptide to polyethylene glycol [75,76] or other carriers. 

Native peptides can also be modified in order to improve their therapeutic potential. As an example, ShK was C-terminally amidated to reduce susceptibility to proteolysis and the oxidation-sensitive methionine residue was replaced by norleucine [70].

Disulfide bridges in peptides also present challenges. It has been shown that they are susceptible to reduction in certain extracellular environments such as the blood [77]. To overcome this potential problem, disulfides can be replaced with diselenium or dicarba bridges, which are less susceptible to degradation. Compared with the sulfur-sulfur bond in disulfide bridges, selenium-selenium bond lengths in diselenium bridges are slightly longer (*ca* 2.02 Å) [77], while the carbon-carbon bond lengths in dicarba bridges are shorter (*ca* 1.34 Å) [78]. In separate studies, diselenium and dicarba bonds have been incorporated in the α-conoxtoxin ImI [77,78]. For both substitutions, the conformation of the analogue was close to that of the native structures, with slight local differences in the vicinity of the substituted residues arising from the different covalent geometries. Nevertheless, biological activity against the nicotinic acetylcholine receptor was retained for both modified peptides, supporting their future use in the design of stable scaffolds for drugs. Recently, it was shown that the introduction of diselenide bridges into α-conoxtoxins enhanced folding efficiency and gave similar or improved potency against nicotinic acetylcholine receptors [79]. In addition, replacing individual disulfide bridges with diselenide bridges in μ-SIIIA did not affect its ability to block Na_V_1.2 [80]. 

Another option for replacing disulfides is to introduce the non-protein amino acid lanthionine, which can be thought of as two alanine residues crosslinked at their β-carbons by a thioether linkage. The monosulfide bridge of lanthionine affords more constrained peptide structures with greater stability toward enzymatic degradation than their disulfide-bridged counterparts [81,82]. However, the presence of only one sulfur in lanthionine, in contrast to the two found in a disulfide bridge, dictates that it is not isosteric with cystine.

Another potential problem associated with disulfide-bridged peptides is the need to ensure formation of the correct linkages in either the synthetic or recombinant product. As reviewed elsewhere [83], some toxins undergo oxidative refolding very efficiently whereas others give quite low yields of native toxin. The efficiency of oxidative refolding is certainly one contributor to the cost of producing GMP-grade peptides for clinical evaluation. Traditionally, the cost of goods has been an issue that has hampered the acceptance of peptides as valuable therapeutic leads, but the dramatic reduction in the cost of synthetic peptides over the past decade has ameliorated this problem to some extent [62].

One strategy to avoid the cost of synthesizing peptides such as μ-conotoxins is to express them in a heterologous system. Bacterial systems are usually preferred, but over-expressed toxins often form inclusion bodies, in which case it is necessary to solubilize the expressed product under denaturing and reducing conditions and then refold it *in vitro* [84]. Alternatively, folded toxins can be obtained by bacterial expression of soluble fusion proteins from which they are released by protease cleavage [85,86,87]. Yeast offers the advantage that the desired protein can be secreted with native disulfide bonds already formed [88,89]. Other useful expression systems are also available [90].

### 5.2. Truncated and Stabilized Peptides

As outlined in the previous section, strategies exist for overcoming many of the potential limitations of peptides as drugs. Nonetheless, these limitations provide an incentive to develop alternatives to naturally occurring peptides, especially those containing several disulfide bridges, as is the case for the μ-conotoxins of interest here. The structural and functional studies on μ-SmIIIA, μ-SIIIA and μ-KIIIA described above provide us with an emerging picture of structural requirements of these peptides as blockers of VGSCs. An important conclusion is that the number of residues in the first loop has no significant effect on the overall conformations of the second and third loops that present critical residues. A second is that the μ-conotoxin scaffold can accommodate backbone spacers [60] and/or removal of individual disulfide bridges [40,91] without compromising bioactivity, emphasizing the adaptability of the μ-conotoxin scaffold and making it a valuable template for peptide engineering. It seems that the N-terminal region could be truncated to produce a minimized analogue that retains the key residues on an α-helical scaffold.

It is well known that helical regions of native peptides or proteins are unlikely to retain their helical structure as isolated peptide fragments, free of the structural constraints imposed by the parent polypeptide or protein. Given that the key residues of several μ-conotoxins have been shown to reside in an α-helix, it is therefore appropriate to consider ways in which truncated analogues of this region could be stabilized in a helical conformation. Several unnatural amino acids have been employed to promote helix formation in peptides, including the α,α-substituted aminoisobutyric acid (Aib) and straight-chain aliphatic amino acids such as aminobutyric acid (Abu), norvaline (Nva) and noeleucine (Nle) [92], but their effects are usually moderate.

**Figure 4:**
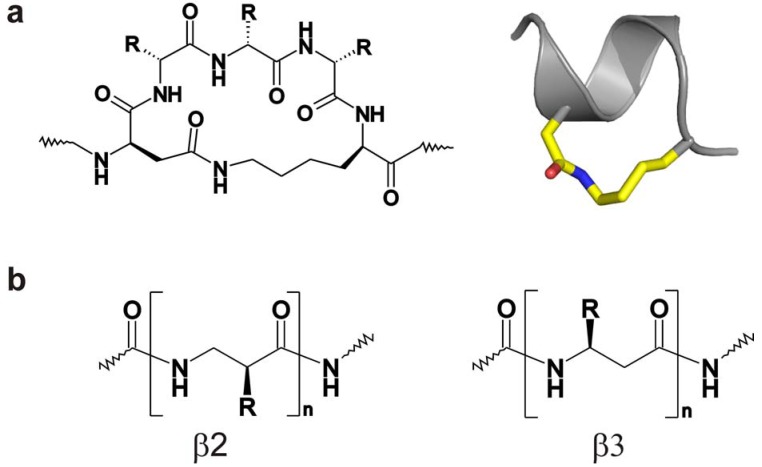
(a) Left hand side: Schematic of *i* to *i *+ 4 lactam bridge linking Asp and Lys side chains. Right hand side: model of helical peptide containing this lactam bridge. **(b)** Schematics of peptides containing β2 (left) and β3 (right) amino acids.

One well-established approach to such stabilization is the introduction of lactam bridges that covalently link carboxylate side chains such as Asp or Glu to ammonium-containing side chains such as Lys or Orn (Figure 4) [92,93,94,95]. There are many examples of the successful stabilization of helical structure in peptides by the introduction of single or multiple lactam bridges [96,97,98,99,100,101,102]; the latter can be positioned sequentially along the peptide sequence or may overlap with one another. Preliminary data suggest that this approach is viable for stabilizing the helical region of the μ-conotoxins of interest here (Khoo, K.K., Bulaj, G. and Norton, R.S., unpublished results).

Other types of ‘helix staples’ have also been described [103,104]. Covalent links between positions *i* and *i * + 7 are very effective [105,106], although not as widely used as *i* to *i *+ 4 staples. Indeed, a recent report demonstrated that a hydrocarbon-stapled peptide not only retained its helical structure and target binding affinity, but was also able to penetrate cells and inhibit the transcription factor NOTCH [107]. 

An exhaustive catalogue of methods for stabilizing helical structure in linear peptides is beyond the scope of this review, but we make note in closing of one additional method for achieving this goal which shows considerable promise, namely the inclusion of β-amino acids. Relative to α-amino acids, β-amino acids have an additional methylene groups inserted in the peptide backbone between C^α^ and either the amide nitrogen (β^2^) or the carbonyl carbon (β^3^) (Figure 4). Various helical structures can be formed by peptides composed of β^2^ or β^3^ amino acids, with β^3^-containing peptides having the same L-configuration as α-helices [108,109,110]. Peptides composed of both α- and β-amino acids and adopting helical structures in solution have been exploited for a number of applications, including the generation of protease-resistant antagonists of anti-apoptotic proteins [111,112].

### 5.3. Peptide Mimetics

An entirely different approach to mimicking helical segments of the µ-conotoxins is to create peptidomimetics that present key functional groups in the same orientation as in the parent peptide but are no longer based on a recognizable peptide scaffold. This strategy has enjoyed some success in mimicking polypeptide toxins [113,114,115,116], although relatively few examples exist because it is a deceptively difficult task to create synthesizable and drug-like scaffolds of discontinuous binding epitopes that are conformationally appropriate and biologically active [117]. 

**Figure 5:**
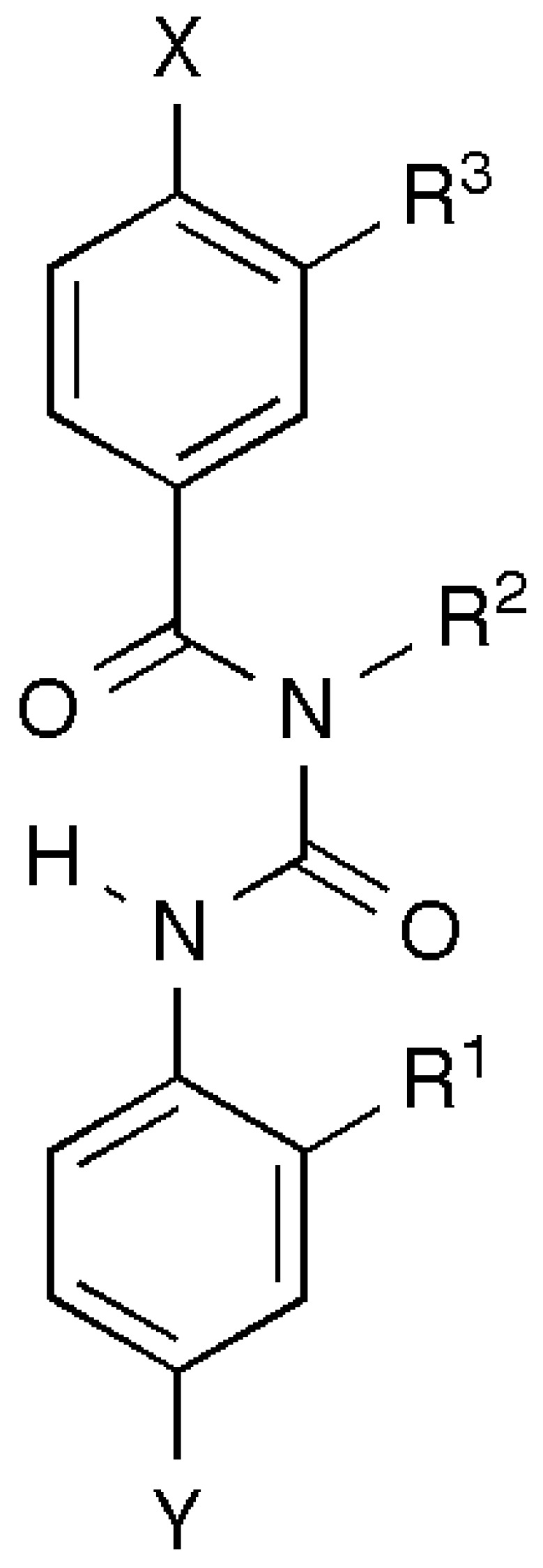
Schematic of a helical mimetic scaffold. This shows a benzoylurea scaffold decorated with three substituents, R^1^, R^2^ and R^3^, which could mimic key functional side chains from the helical region of a µ-conotoxin such as µ-KIIIA or µ-SIIIA. X and Y can be any substituent, including additional aromatic rings.

Various non-peptidic scaffolds have been developed to mimic helices. The terphenyl scaffold designed by the Hamilton group stands out in terms of simplicity of design and synthesis of non-peptide, three-residue mimetics of the discontinuous binding epitope representing one face of an α-helix [118], and has been used to develop a mimetic of the pro-apoptotic α-helical Bak peptide as an inhibitor of the Bak/Bcl-xL interaction [119] as well as a mimetic of an α-helical hydrophobic peptide that inhibits the assembly of a six-helix bundle corresponding to the fusion-active conformation of the HIV gp41 protein [120]. This group has also developed other helical mimetic scaffolds, including a more water soluble 5-6-5 imidazole-phenyl-thiazole [121]. Recently, the benzoylurea scaffold (Figure 5) has been introduced as a basis for mimetics with improved solubility and synthetic feasibility [122,123].

Our approach to generating non-peptidic analogues of the µ-conotoxins will be to target the three most important residues for VGSC affinity and subtype specificity. Attempting to target more than three residues can lead to difficult design and synthetic issues while targeting fewer is unlikely to give a meaningful biological readout. Our experience has been that low µM and selective activity are readily attainable in mimetic triads. Options for improving activity include subsequent extension to mimic a fourth residue or treating the mimetic triad as a “hit” and subjecting it to hit-to-lead medicinal chemistry optimization. 

## 6. Conclusions

There is ample evidence that VGSCs, especially Na_V_1.3, Na_V_1.7 and Na_V_1.8, are excellent targets for the treatment of pain. Moreover, there is equally compelling evidence that the µ-conotoxins are potent blockers of VGSCs, although in most cases with Na_V_1 selectivity profiles (if known) that are broader than desirable in therapeutic leads. The challenges now are to gain a greater understanding of the exact chemical and conformational requirements for both potent and selective VGSC blockade, and to apply that knowledge to the development of peptidic as well as peptidomimetic analogues of the native toxins, with the goal of developing novel analgesics for the treatment of chronic pain.
